# Sphingosine 1-phosphate to p38 signaling via S1P_1_ receptor and Gαi/o evokes augmentation of capsaicin-induced ionic currents in mouse sensory neurons

**DOI:** 10.1186/1744-8069-10-74

**Published:** 2014-11-28

**Authors:** Michiel Langeslag, Serena Quarta, Michael G Leitner, Michaela Kress, Norbert Mair

**Affiliations:** Division Physiology, DPMP, Medical University Innsbruck, Fritz-Pregl-Str. 3-I, 6020 Innsbruck, Austria; Department of Neurophysiology, Institute for Physiology and Pathophysiology, Philipps University of Marburg, Marburg, Germany

**Keywords:** Sphingosine 1-phosphate, TRPV1, Capsaicin, Gαi, Phosphoinositide 3-kinase, MAP-kinase p38

## Abstract

The perception of painful thermal stimuli by sensory neurons is largely mediated by TRPV1. Upon tissue injury or inflammation, S1P is secreted by thrombocytes as part of an inflammatory cocktail, which sensitizes nociceptive neurons towards thermal stimuli. S1P acts on G-protein coupled receptors that are expressed in sensory neurons and sensitize TRPV1 channels towards thermal stimuli. In this study, the S1P mediated signaling pathway required for sensitization of TRPV1 channels was explored.

The capsaicin induced peak inward current (I_CAPS_) of sensory neurons was significantly increased after S1P stimulation within minutes after application. The potentiation of I_CAPS_ resulted from activation of Gαi through G-protein coupled receptors for S1P. Consequently, Gαi led to a signaling cascade, involving phosphoinositide-3-kinase (PI_3_K) and protein kinase C, which augmented I_CAPS_ in nociceptive neurons. The S1P_1_ receptor agonist SEW2871 resulted in activation of the same signaling pathway and potentiation of I_CAPS_. Furthermore, the mitogen-activated protein kinase p38 was phosphorylated after S1P stimulation and inhibition of p38 signaling by SB203580 prevented the S1P-induced I_CAPS_ potentiation. The current data suggest that S1P sensitized I_CAPS_ through G-protein coupled S1P_1_ receptor activation of Gαi-PI_3_K-PKC-p38 signaling pathway in sensory neurons.

**Electronic supplementary material:**

The online version of this article (doi:10.1186/1744-8069-10-74) contains supplementary material, which is available to authorized users.

## Background

The perception of pain is mediated by nociceptive primary afferent neurons that are excited upon painful thermal, mechanical or chemical stimuli [1]. These nociceptive neurons demonstrate increased sensitivity towards painful stimuli during inflammation or injury when challenged by pro-inflammatory mediators (e.g. bradykinin, prostaglandin) [2, 3]. The cellular and molecular mechanisms that are involved in thermal pain perception and sensitization are well studied and comprise many different signaling pathways and proteins [4, 5].

The perception of heat involves members of the transient receptor potential (TRP) ion channels, more specifically members of the vanilloid subfamily (TRPV). In particular activation of TRPV1 ion channels results in the excitation of nociceptors and consequently the perception of pain [6, 7]. TRPV1 is a non-specific cation channel that is not only activated by heat but also by vanilloid agonists like capsaicin and resiniferatoxin, by low pH (<5.5) and various lipids [8, 9]. The activation of TRPV1 ion channels results in opening of the channel and subsequent membrane depolarization of nociceptive neurons.

In the presence of inflammatory mediators, the threshold temperature at which TRPV1 channels are activated is decreased and nociceptive neurons respond to thermal stimuli at lower temperatures and with an augmented response. The regulation of TRPV1 by inflammatory mediators released by the immune system receives extensive attention since it is clinically relevant for developing pathological and chronic pain. Activation of G-protein coupled or tyrosine kinase receptors modulate TRPV1 ion channel activity via various intracellular signaling pathways [10, 11].

Tissue damage that usually coincides with damage to the blood vessels results in tissue invasion of different cells of the immune system together with thrombocytes. At the injury site, thrombocytes are activated and secrete a variety of immunomodulatory compounds including the sphingolipid sphingosine 1-phosphate (S1P). S1P can activate signaling pathways either through diffusion over the plasmamembrane or through binding to S1P specific receptors (S1P_1–5_) at the plasmamembrane.

After binding of S1P to its specific receptors, activation of the receptor subtype determines the heteromeric G-protein signaling pathway. For example, the S1P_1_ receptor solely signals through Gαi-proteins whereas the S1P_3_ receptor can activate Gαi, Gαq and/or Gα12/13 signaling pathways. Through this pleiotropic activation, S1P can exert its effects on various signaling pathways involving e.g. Rho, PLC, p38 and ERK (p42/44) signaling [12]. Previously we have shown that nociceptors primarily express S1P_1_ and S1P_3_ receptors whereas the larger NF200-positive cells express S1P_2_ receptors. Recently it has been found that S1P enhances neuron excitability [13, 14] and sensitizes dorsal root ganglion (DRG) neurons to heat [15].

Converging evidence from pharmacological and genetic models suggests that the S1P_1_ receptor is a main contributor to S1P-induced hyperexcitability and heat sensitization in mouse nociceptors [14–16]. Although S1P_1_ receptor signaling is restricted to Gαi-mediated signal transduction, the molecular players of TRPV1 mediated sensitization by S1P remain unclear. Here we explore the S1P-PI_3_K-p38 signaling pathway in sensory neurons for the potentiation of capsaicin-induced, excitatory inward currents.

## Results

### S1P-induced potentiation of capsaicin-activated excitatory inward currents

**Figure 1:**
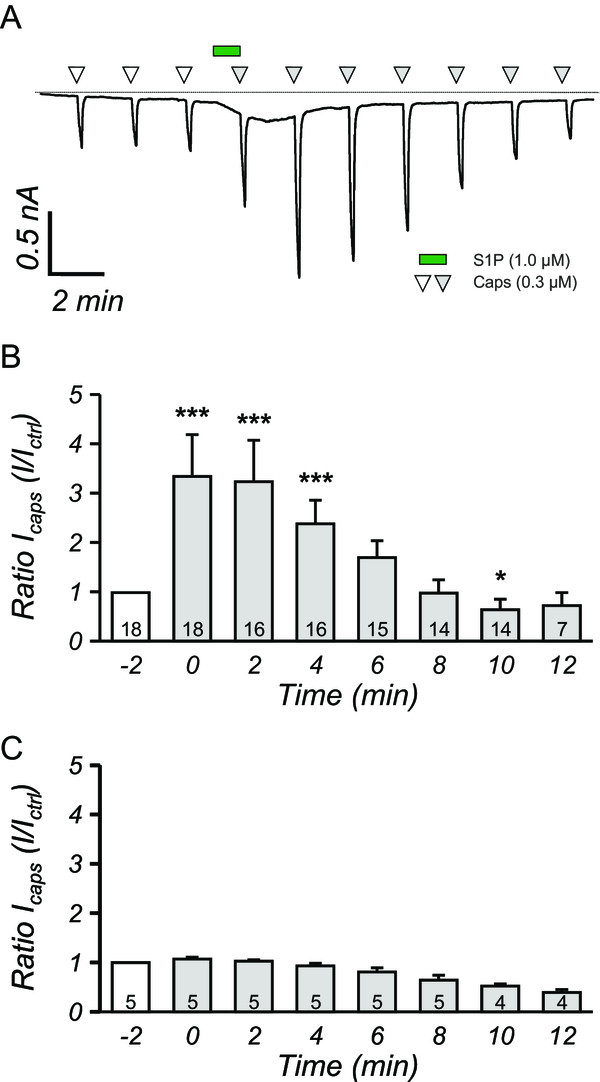
**S1P potentiates capsaicin-induced currents in cultured DRG sensory neurons. A**, a typical voltage-clamp recording from a DRG neuron that is repetitively stimulated with capsaicin (0.3 μM white and grey triangles). Between the 3^rd^ and 4^th^ capsaicin application DRG neurons were stimulated with 1.0 μM S1P (green bar) that resulted in augmentation of I_CAPS_ (grey triangles). The dashed line represents 0 nA. **B**, Quantification of S1P induced potentiation of I_CAPS_, which were normalized to amplitude of the 3^rd^ I_CAPS_, showed that S1P potently increased I_CAPS_ up to 4 minutes after S1P application. ***p < 0.001, *p < 0.05, MWU-test. **C**, Repetitive application of capsaicin did not result in potentiation of I_CAPS_. Numbers within the bars represent the number of individual recorded.

In humans and mice, the bio-active lipid S1P evokes spontaneous pain behavior [17]. Besides, intradermal injection of S1P in the hindpaw of mice induces heat-hypersensitivity as indicated by reduction of reflex paw withdrawal latencies in response to radiant heat stimulation [15]. Responses of nociceptive neurons to heat are mainly mediated by the transient receptor potential vanilloid receptor TRPV1 that is essential for the development of thermal hypersensitivity [6, 18, 19]. To assess TRPV1 function we performed whole-cell recordings of capsaicin-evoked excitatory inward currents (I_CAPS_, 0.3 μM capsaicin) from neurons isolated from mouse dorsal root ganglia (DRG). Application of S1P (1.0 μM, 60s) caused a significant increase of I_CAPS_ peak amplitudes (fold increase: 3.22 ± 0.81, n = 18, p < 0.001, Figure 1A,B). The potentiation of I_CAPS_ was transient and fully recovered within 6 minutes, suggesting a modification of ion channel function as underlying mechanism. Repetitive application of capsaicin did not result in potentiation of the inward current in DRG neurons for the first five to six applications (Figure 1C) and obviously was prone to desensitization at later time points.

**Figure 2:**
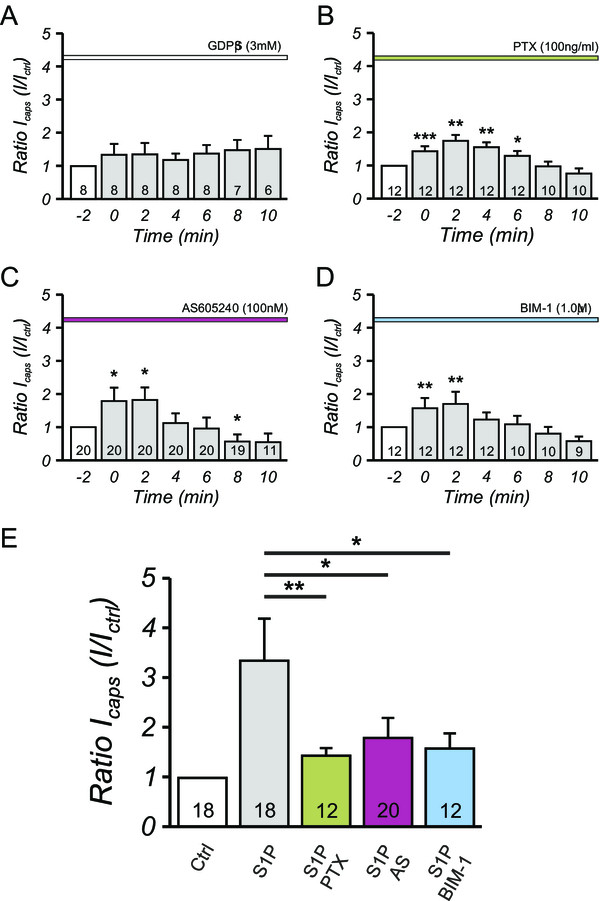
**Inhibition of downstream signaling mediated by S1P largely prevents potentiation of I**_**CAPS**_ **in sensory neurons. A**, inhibition of heteromeric G-proteins by inclusion of 3.0 mM GDPβS in the patch pipette fully prevented the potentiation of I_CAPS_ mediated by 1.0 μM S1P. Furthermore, inhibition of Gαi by pertussis toxin (100 ng/ml PTX, **B**), PI_3_Kγ (100nM AS605240, **C**) and PKC (1.0 μM BIM-1, **D**) decreased the amplitude of S1P mediated I_CAPS_ potentiation in sensory nociceptors significantly compared to the enhanced I_CAPS_ after S1P stimulation **(E)**. ***p < 0.001, **p < 0.01, *p < 0.05, MWU-test, numbers within the bars represent the number of individual cells recorded.

To explore whether S1P potentiated capsaicin induced currents through G-protein coupled receptors (GPCR) activation or rather by direct activation of the channel. In the presence of extracellular suramin (100 μM), which is generally accepted to uncouple heteromeric G-proteins from their ligand-binding receptor subunit, S1P failed to evoke an increase in I_CAPS_ (see Additional file 1). To more specifically prevent G-protein mediated signaling, GDPβS (3.0 mM) was included in the intracellular solution. In nociceptive neurons treated with GDPβS, S1P no longer induced an increase in I_CAPS_ (Figure 2A). Therefore it is concluded that S1P acted on a G-protein coupled receptor (GPCR) to regulate TRPV1-induced currents.

### S1P potentiates I_CAPS_ via Gαi-PI_3_K-PKC signaling cascade

Whereas S1P_2_ and S1P_3_ receptors couple to all three G-protein types [20, 21], S1P_1_ exclusively couples to Gαi/o and no activation of other heteromeric G-proteins has been reported to date [22, 23]. We therefore pretreated sensory neurons for 20 hours with the Gαi/o-selective inhibitor pertussis toxin (PTX; 100 ng/ml). The potentiation of I_CAPS_ after conditioning stimulation with S1P was reduced by pretreatment with PTX in the patch-clamp recordings, suggesting the involvement of Gαi/o heteromeric G-proteins in the signaling process (Figure 2B). It is generally accepted that activation of Gαi/o α-subunit inhibits the adenylyl cyclase/protein kinase A (PKA) pathway. Since TRPV1 activity is upregulated via PKA mediated phosphorylation, a S1P-dependent reduction of PKA activity should attenuate heat responses in sensory neurons [24–28]. However, as shown above, S1P_1_ augmented nociceptor activity in three independent models. This apparent controversy could be explained if S1P activated a sensitizing pathway via β/γ G-protein subunits rather than activation via the α-subunit. Phosphatidyl-inositol-triphosphate kinase (PI_3_K) isoenzymes have been identified as targets of S1P_1_ and S1P_3_ signaling in some studies [29–31]. After inhibition of the phosphoinositide kinases PIP_4_K and PI_3_K by Wortmannin (1.0 μM, data not shown), S1P did not facilitate I_CAPS_ to the same extent as in control ECS. Selective inhibition of PI_3_K by AS605240 (100nM), also attenutated S1P potentiation of I_CAPS_and the inhibition of S1P-induced potentiation of I_CAPS_ by Wortmannin and AS605240 was comparable to the decrease in S1P-mediated TRPV1 potentiation after PTX incubation (Figure 2E).

Since PI_3_K can activate PKCs [32], pretreatment with the non-selective PKC inhibitor bisindolylmaleimide-1 (BIM-1, 1.0 μM) was used to investigate whether this downstream signaling pathway was involved in S1P induced modification of I_CAPS_. After inhibition of PKC, I_CAPS_ was still potentiated after conditioning exposure to S1P although the degree of sensitization was significantly reduced. In comparison to the S1P-mediated potentiation, BIM-1 potently attenuated the potentiation of I_CAPS_ induced by S1P in patch-clamp recordings of DRG neurons (Figure 2D,E). The inhibition of S1P-mediated I_CAPS_ potentiation was to the same extent as observed after inhibition of the Gαi and PI_3_K pathway (Figure 2E).

### SEW2871 induced S1P_1_ receptor activation sensitizes I_CAPS_

**Figure 3:**
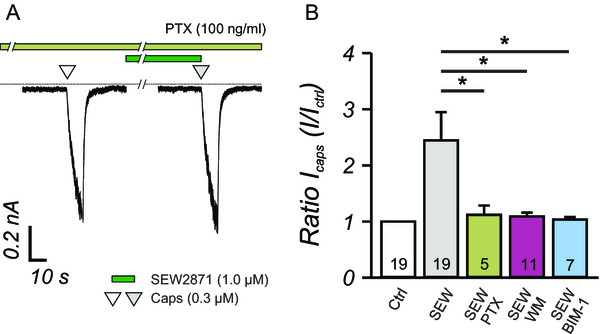
**I**_**CAPS**_ **sensitization by depends on S1P**_**1**_ **receptor signaling. A**, The enhancement of I_CAPS_ by the S1P_1_ specific receptor agonist SEW2871 (1.0 μM) was completely inhibited by pertussis toxin (PTX, 100 ng/ml). The dashed line signifies the zero current. **B**, Moreover, inhibition of the PI_3_K by Wortmannin (WM, 1.0 μM) and PKC (BIM-1, 1.0 μM) fully prevented the potentiation of I_CAPS_ that was induced by S1P_1_ receptor activation by SEW2781 (SEW). *p < 0.05, MWU-test, numbers within the bars represent the number of individual cells recorded.

Stimulation of DRG neurons with S1P could activate both S1P_1_ and S1P_3_-receptors on the same neuron. The S1P_1_-receptor expressed on nociceptive neurons has been shown to be the major signaling pathway contributing to heat sensitization and presumably affected nociceptive neurons directly [15]. Previously we have shown that SEW2871, a specific and selective agonist for the S1P_1_ receptor, also facilitates capsaicin-induced currents [15]. Since S1P_1_ receptor only signals via Gαi, we used SEW2871 to assess the involvement of the Gαi-signaling pathway in S1P mediated TRPV1 potentiation (Figure 3A). The sensitization of I_CAPS_ by SEW2871 (1.0 μM) was completely inhibited by 20 h pre-incubation with PTX and thus completely relied on the activation of Gαi (Figure 3A,B). In line with earlier findings in this study, inhibition of phophoinositide kinases by Wortmannin or PKCs by BIM-1 fully prevented potentiation of capsaicin-induced inward currents by SEW2871. Accordingly, these data suggested that Gαi-PI_3_K-PKC signaling was activated through the S1P_1_ pathway solely, whereas additional S1P receptors might play a minor role in S1P-mediated I_CAPS_ sensitization.

### Activation of MAP-kinase p38 by S1P increases I_CAPS_

**Figure 4:**
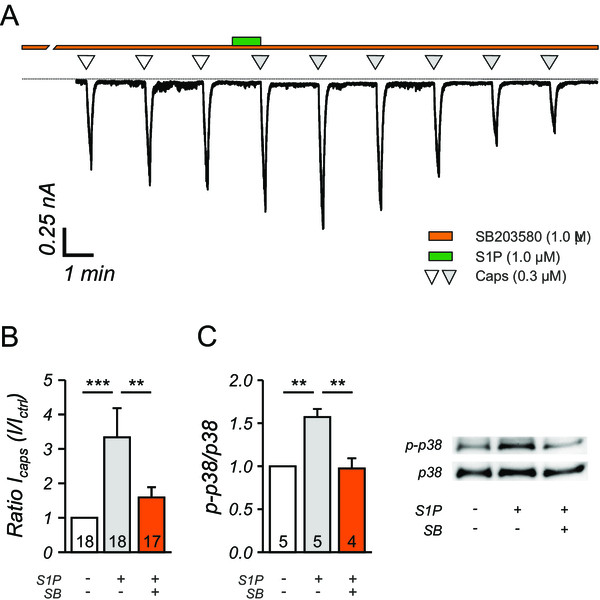
**Activation of p38 is critical in the S1P induced I**_**CAPS**_ **potentiation in sensory neurons. A**, In a typical current recording of a nociceptor, the I_CAPS_ increase caused by 1.0 μM S1P stimulation is greatly diminished by inhibition of p38 activation by SB203850 (1.0 μM). Zero current is drawn as dashed line. **B**, Pretreatment of sensory DRG neurons with SB203850 (1.0 μM) significantly reduced the S1P mediated potentiation of I_CAPS_. ***p < 0.001, **p < 0.01, MWU-test. **C**, Western blot analysis of p38 activation (p-p38) showed that S1P significantly increased the p-p38 levels in sensory neurons, whereas SB23850 (SB) reversed the p-p38 levels to control, unstimulated levels. ***p < 0.001, **p < 0.01, ANOVA on Ranks followed by Tukey post-hoc test, numbers within the bars represent the number of individual cells recorded.

S1P receptor signaling pathways activates p38 or p42/44 MAP kinases through phosphorylation [33, 34]. Therefore, the levels of phosphorylated p42/44 and p38 were assessed by Western blot with phosphorylation-specific antibodies and subsequent quantification of the blots. We did not detect significant activation of the MEK/ERK pathway by S1P as the relative amount of p-p42/p44 phosphorylation was not changed (data not shown). Interestingly however, p-p38 levels increased significantly (1.57 ± 0.09, n = 5) following S1P stimulation of neurons cultures, which suggested a major contribution of p38 in the S1P signaling pathway (Figure 4C). The phosphorylation of p38 induced by S1P was completely inhibited by SB203580 (0.97 ± 0.12, n = 4), a specific inhibitor of the p38 signaling pathway (Figure 4C). In line with this finding, the potentiation of I_CAPS_ evoked by S1P was largely inhibited after pre-incubation with SB203580 (Figure 4A). The I_CAPS_ recorded immediately after S1P stimulation was significantly reduced compared to control conditions (Figure 4B, Ctrl; 3.22 ± 0.81 vs. SB203850; 1.59 ± 0.30, p < 0.01, MWU). Overall, the SB203580-mediated inhibition of the I_CAPS_ potentiation was similar to the application of by PTX, AS605240 and BIM-1.

## Discussion

Our study revealed a prominent role for the S1P_1_-Gαi-signaling pathway to enhance capsaicin-induced inward currents. This signaling pathway contributed to the S1P induced increase in nociceptors responsiveness to capsaicin and presumably also to thermal stimuli. We characterized the S1P-induced signaling pathway that contributed to enhanced TRPV1 activity and involved a heteromeric Gαi-protein, PI_3_K, activation of PKC and MAPK p38.

To date, five members of the metabotropic Edg G-protein coupled receptor (GPCR) family are known to specifically bind S1P and to regulate inflammatory and regenerative processes in various systems [35, 36]. Crosstalk of cytokines, growth factors and S1P in inflamed tissue occurs and several reports have addressed the expression of S1P_1_ and S1P_3_ receptors in primary afferent nociceptors that sense inflammatory mediators and respond more vigorously to natural stimuli upon inflammation [13, 15–17, 37]. Therefore, a major component of nociceptor sensitization that is associated with inflammation could be mediated by S1P acting via S1P_1_ and/or S1P_3_ mediated signaling pathways. The widespread expression of S1P_3_ receptors in all classes of sensory neurons suggests the S1P_3_ receptor as a candidate to regulate the sensitivity of multiple sensory modalities. Indeed activation of S1P_3_ receptors in nociceptors induces a pain-like behavior in human and mice. This pain-like behavior is evoked by a membrane depolarization involving S1P_3_ receptors [17].

A smaller portion of sensory peptidergic and non-peptidergic neurons express S1P_1_ receptors [15]. S1P induces thermal hypersensitivity in mice and enhances the excitability of sensory neurons [13, 15]. In a transgenic mouse where S1P_1_ receptors are conditionally ablated in Nav1.8 expressing neurons, the S1P induced heat hypersensitivity is attenuated [15]. In line with these reports, the enhancement of TRPV1 activity following S1P conditioning was mimicked by the S1P_1_ agonist SEW2871. The augmentation of I_CAPS_ by S1P and SEW2871 was inhibited in the presence of PTX that selectively interferes with the inhibitory G-protein Gαi. This suggests that S1P_1_ utilizes Gαi for signal transduction in nociceptors, just like in other tissues.

Although the contribution of Gαi to S1P_1_ receptor signaling is well accepted it is largely unknown which downstream intracellular signaling pathways are activated in nociceptive neurons. In general, the S1P_1_ receptor has been associated with three different signaling pathways downstream of Gαi-proteins. The classical Gαi signaling involves inhibition of adenylate cyclase and reduction of cyclic AMP levels or inhibition of ERK signaling within the cell [38, 39]. Since cAMP/PKA signaling increases the open probability of TRPV1 ion channels, it can be anticipated that decreased cAMP levels rather leave TRPV1 in a less sensitive state and reduces I_CAPS_ amplitudes which would oppose our findings.

The signaling cascade for S1P induced facilitation of capsaicin-induced currents also differs from the Gαi-mediated activation of ERK1/2 through which S1P_1_ receptors regulate embryonic self-renewal [40]. TrkA receptor-mediated ERK1/2 acyivation (p42/44) by nerve growth factor induces heat hypersensitivity and potentiates nociceptor responses to capsaicin [41–43]. However, ERK1/2 activation was not induced in nociceptive neurons upon S1P stimulation. Therefore the observed enhancement of I_CAPS_ is unlikely to be explained by these two classical Gαi signaling pathways.

An alternative S1P_1_ signaling pathway involves Gαi-mediated activation of PI_3_K that has been reported to mediate chemotaxis of natural killer cells [44]. Furthermore PI_3_K signaling pathways have been associated with the regulation of TRPV1 [45] and increasing evidence suggests that particular isoforms of PI_3_K signal downstream of GPCRs [46]. In our hands, the potentiation of I_CAPS_ through S1P_1_ largely depended on PI_3_K. Thus our results link PI_3_K as novel component of GPCR signaling for the regulation of nociceptor heat hypersensitivity. Downstream of PI_3_K several kinases of the mitogen activated protein kinase (MAPK) family can be activated. In particular activation of MAP kinase p38 is strongly associated with Gαi/PI_3_K signaling through S1P receptors [34, 47]. The MAPK p38 functions as a mediator of cellular stresses such as inflammation and apoptosis [48, 49]. Besides, recent findings showed that activation of p38 contributes to the development and maintenance of both neuropathic and inflammatory pain: For example, in mouse models of neuropathic pain, activation of p38 is observed in microglial cells in the spinal cord and in DRG neurons [50–52]. Activation of p38 can have pleiotropic effects within cells, and in sensory neurons it can lead to increased current density of Nav1.8 channels by phosphorylation on short-term [53] and upregulation of Nav1.3 channels on the long-term [54]. The multimodal transducer ion channel TRPV1 is activated by noxious heat and capsaicin the pungent ingredient of hot chili peppers, and it plays a major role in the generation of heat hypersensitivity as occurs as a consequence of tissue inflammation [6, 18, 19, 55]. The sensitivity of TRPV1 to heat and capsaicin depends on the phosphorylation status of the channel at intracellular serine/threonine or tyrosine sites [24, 26, 56]. The degree of serine or threonine phosphorylation at specific consensus sites, e.g. for PKC, regulates the TRPV1 channel activity. Serine phosphorylation determines the open probability of the individual TRPV1 channel complex [8, 10]. Besides, modification at tyrosine residues regulates insertion of preformed TRPV1 complexes into the cellular membrane [43]. Numerous inflammatory mediators including S1P, which target to metabotropic receptors in the nociceptor membrane, are able to facilitate I_CAPS_ either by activating tyrosine kinases or protein kinases A or C. The fast changes in capsaicin sensitivity of the sensory neurons that occurred within 1 minute of S1P stimulation are likely attributable to phosphorylation of the already existing TRPV1 channels in the cell membrane. Some of the phosphorylation sites at intracellular domains of the TRPV1 channel protein do not show preference for PKC, PKA ,or CaMKII (calcium/calmodulin dependent protein kinase II) and could be possible targets for p38 MAP kinase phosphorylation [57]. Although the present experiments and a recent report which links PI_3_K/p38 and TRPV1 [45] do not unequivocally demonstrate the mutual interdependence of these kinase systems, p38 activation downstream of PI_3_K is likely and in line with ample evidence from other systems [33, 34, 45, 47].

Therefore we suggest that S1P_1_ receptor activation and downstream Gαi to PI_3_K to p38 signaling as important components for the S1P induced nociceptive hypersensitivity towards thermal stimuli. S1P_1_ to p38 mediated sensitization of TRPV1 may play a role in the early initiation phase of heat hypersensitivity in mice.

## Materials and methods

### Ethic statement

All animal breeding and experiments have been performed with permission of the Austrian BMWF ministry (BMWF-66.011/0113-II/3b/2010; BMWF-66.011/0051-II/10b2008; GZ 66.011/85-C/GT/2007) and according to ethical guidelines of the IASP (International Association for the Study of Pain).

### Primary sensory neuron culture

Lumbar DRG containing the cell bodies of primary afferents that project into the hindpaw were harvested from adult male mice (age 8–16 weeks) as previously published [58, 59]. After removal of the connective tissue, ganglia were incubated in Liberase Blendzyme 1 (9 mg/100 ml DMEM, Roche) for 2 times 30 min. After washing with PBS (PAA), 1× Trypsin-EDTA (Invitrogen) was added for 15 min. and DRG were washed with TNB™ medium (Biochrom) supplemented with L-glutamin (Invitrogen), penicillin G sodium, streptomycin sulfate (Invitrogen), and Protein-Lipid-Komplex™ (Biochrom). The DRG were dissociated with a fire-polished Pasteur pipette and centrifuged through a 3.5% BSA gradient (Sigma) to eliminate non-neuronal cells. The sensory neurons were resuspended, plated on coverslips coated with poly-L-Lysine/laminin-1 (Sigma), and cultivated in supplemented TNB™ containing mNGF 2.5S (Alomone Labs, 10 μg/100 ml TNB-medium) at 37 °C in 5% CO2 for 24–36 h.

### Patch-clamp recordings

Using the whole-cell voltage-clamp configuration of the patch-clamp technique, ionic currents were recorded from isolated neurons at -80 mV holding potential after 18–32 hours as previously published [15, 58]. The external solution (ECS) contained (in mM): 145 NaCl, 5 KCl, 2 CaCl_2_, 1 MgCl_2_ (all Sigma), 10 glucose and 10 HEPES (Merck, Darmstadt, Germany), at pH 7.3 adjusted with NaOH (Merck). Borosilicate glass micropipettes (Science Products, Hofheim, Germany) pulled with a horizontal puller (Sutter Instruments Company, Novato, CA, USA) were filled with internal solution (ICS, in mM): 148 KCl, 2 MgCl_2_, 2 Na-ATP, 0.2 Na-GTP, 0.1 CaCl_2_, 1 EGTA (all Sigma) and 10 HEPES (Merck), at pH 7.3 adjusted with KOH (Merck). After filling, electrode resistance was 4–6 MΩ. Currents were filtered at 2.9 kHz, sampled at 3 kHz and recorded using an EPC-9 (HEKA, Germany) and the Pulse v8.74 software (HEKA) without Rs compensation. Experiments were performed at room temperature and only one neuron was tested per Petri dish. An automated seven-barrel system with common outlet positioned at 100 μm distance from the recorded cell was used for fast drug administration [15]. S1P (1.0 μM) was used as intermittent conditioning stimuli (60s). S1P, capsaicin, PTX and GDPβS were purchased from Sigma Aldrich. All other chemicals were purchased from Merck-Calbiochem.

### Western blot

Sensory neurons were plated on poly-L-lysine/laminin-coated dishes, kept in culture for 24 h and stimulated with 1.0 μM S1P for 5 minutes in ECS or 30 minute pre-treated with 1.0 μM p38 inhibitor SB203580 before S1P stimulation. Cells were harvested in freshly prepared, ice-cold lysis RIPA-buffer (50 mM Tris–HCl, 150 mM NaCl, 50 mM NaF, 5 mM EDTA, 0.5% Deoxycholic Acid, 0.1% SDS, 1% Nonidet P-40, all Sigma). The phosphatase inhibitors sodium-orthovanadate (200 μM) and β-glycerophosphate (40 mM, both Sigma) were added to the RIPA buffer to prevent protein dephosphorylation. A protease-inhibitor cocktail (1:10, Sigma) was used to protect proteins from proteolysis. SDS-PAGE was performed under standard denaturing conditions using hand casted 10% polyacrylamide gels (Mini-PROTEAN, Bio-Rad Laboratories). Equal amounts of protein were loaded to each lane of the gels. Spectra Multicolor Broad Range Protein Ladder (Fermentas) was used as a molecular weight standard. Gels were blotted immediately after electrophoresis onto polyvinylidene fluoride membrane (Hybond-P, GE Healthcare). For immunodetection, membranes were blocked for 1 h with 5% (w/v) BSA and 0.1% (v/v) Tween-20 in Tris-buffered saline, pH 7.6, at room temperature. Antibodies were used according to the manufacturer’s instructions. The following antibodies were used: anti-phospho-p38 and anti-p38 (all Cell Signaling Technology) and peroxidase-conjugated α-rabbit IgG (1:5000; Pierce) as secondary antibody. Visualization of blots was performed with enhanced chemiluminescence by using the SuperSignal West Pico Chemiluminescent Substrate (Thermo Scientific). Membranes were scanned with LAS4000 luminescent imager (GE Healthcare). Quantification was performed using ImageJ software and relative values for phosphorylated proteins are represented as units after normalization to the non-phosphorylated form.

### Statistical analysis

Data are presented as mean ± SEM. For detailed statistical analysis the Sigmastat 3.0 (Aspire Software International) software package was used and Mann Whitney-U test (MWU) or ANOVA on Ranks followed by Tukey post-hoc test were calculated. Differences were considered statistically significant at p < 0.05.

## Electronic supplementary material

Additional file 1: **Suramin prevents S1P-induced I**_**CAPS**_ **sensitization. A**, The augmentation of I_CAPS_ by S1P (1.0 μM) was completely inhibited by extracellular suramin (100 μM) pretreatment. The dashed line signifies the zero current. **B**, The uncoupling of heteromeric G-proteins from the G-protein coupled receptors significantly inhibited S1P-induced I_CAPS_ potentiation in sensory neurons. ***p < 0.001, **p < 0.01, MWU, numbers within the bars represent the number of individual cells recorded. (PDF 255 KB)

## Abbreviations

S1P: Sphingosine 1-phosphate
TRPV1: Transient receptor potential vanilloid 1
DRG: Dorsal root ganglia
I_CAPS_: Capsaicin-activated inward current
PI_3_K: Phosphatidylinositol-3-kinase
PKC: Protein kinase C
MAPK: Mitogen activated protein kinase
GPCR: G-protein coupled receptor
GDPβS: Guanosine 5′-[β-thio]diphosphate
PTX: Pertussis toxin
PKA: Protein kinase A
ECS: Extracellular solution
ICS: Intracellular solution
PBS: Phosphate buffered saline
DMEM: Dulbecco’s modfified Eagle medium
BSA: Bovine serum albumin.
