# Supplementary figures and images for: Sphingosine 1-phosphate to p38 signaling via S1P1 receptor and Gαi/o evokes augmentation of capsaicin-induced ionic currents in mouse sensory neurons
Source: Mol Pain. 2014 Nov 28;10:74. doi: 10.1186/1744-8069-10-74 (PMC4280769; doi:10.1186/1744-8069-10-74)

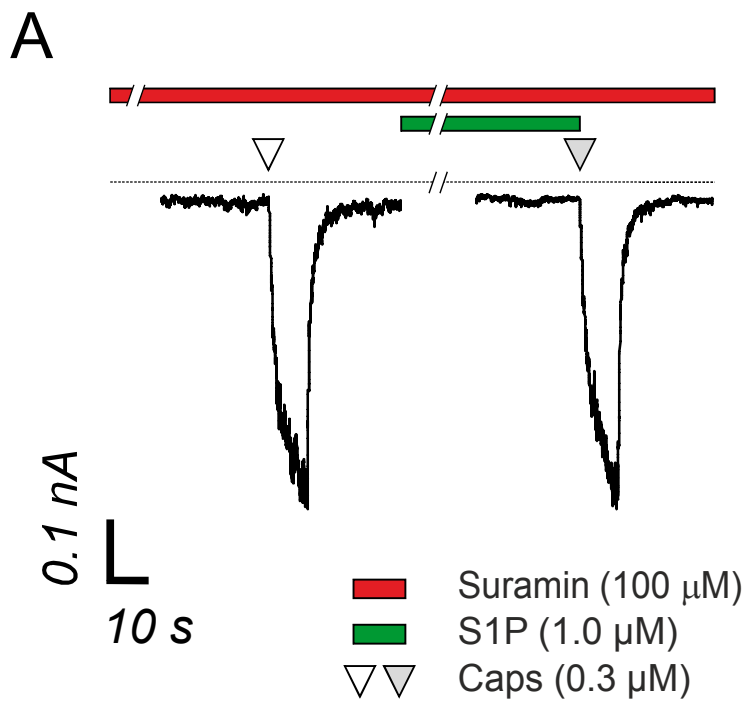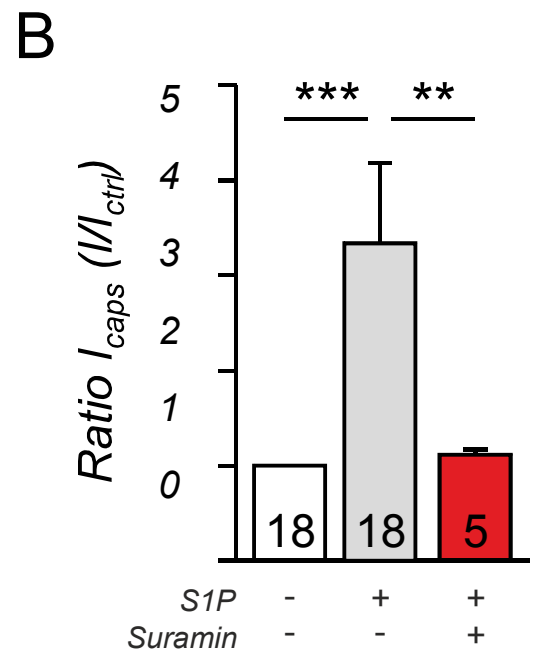

Supplement: Supplementary file 1 — Additional file 1: Suramin prevents S1P-induced I CAPS sensitization. A, The augmentation of ICAPS by S1P (1.0 μM) was completely inhibited by extracellular suramin (100 μM) pretreatment. The dashed line signifies the zero current. B, The uncoupling of heteromeric G-proteins from the G-protein coupled receptors significantly inhibited S1P-induced ICAPS potentiation in sensory neurons. ***p < 0.001, **p < 0.01, MWU, numbers within the bars represent the number of individual cells recorded. (PDF 255 KB) [file 12990_2014_710_MOESM1_ESM.pdf]
